# Higher Impulsivity As a Distinctive Trait of Severe Cocaine Addiction among Individuals Treated for Cocaine or Alcohol Use Disorders

**DOI:** 10.3389/fpsyt.2018.00026

**Published:** 2018-02-13

**Authors:** Nuria García-Marchena, David Ladrón de Guevara-Miranda, María Pedraz, Pedro Fernando Araos, Gabriel Rubio, Juan Jesús Ruiz, Francisco Javier Pavón, Antonia Serrano, Estela Castilla-Ortega, Luis J. Santín, Fernando Rodríguez de Fonseca

**Affiliations:** ^1^Unidad Gestión Clínica de Salud Mental, Instituto de Investigación Biomédica de Málaga (IBIMA), Hospital Regional Universitario de Málaga, Málaga, Spain; ^2^Departamento de Psicobiología y Metodología de las Ciencias del Comportamiento, Instituto de Investigación Biomédica de Málaga (IBIMA), Facultad de Psicología, Universidad de Málaga, Málaga, Spain; ^3^Servicio de Psiquiatría, Instituto de Investigación I+12, Hospital 12 de Octubre, Madrid, Spain; ^4^Centro Provincial de Drogodependencias, Málaga, Spain

**Keywords:** cocaine, alcohol, impulsivity, latent class analysis, psychiatric comorbidity

## Abstract

**Aims:**

Despite alcohol being the most often used addictive substance among addicted patients, use of other substances such as cocaine has increased over recent years, and the combination of both drugs aggravates health impairment and complicates clinical assessment. The aim of this study is to identify and characterize heterogeneous subgroups of cocaine- and alcohol-addicted patients with common characteristics based on substance use disorders, psychiatric comorbidity and impulsivity.

**Methods:**

A total of 214 subjects with cocaine and/or alcohol use disorders were recruited from outpatient treatment programs and clinically assessed. A latent class analysis was used to establish phenotypic categories according to diagnosis of cocaine and alcohol use disorders, mental disorders, and impulsivity scores. Relevant variables were examined in the latent classes (LCs) using correlation and analyses of variance and covariance.

**Results:**

Four LCs of addicted patients were identified: *Class 1* (45.3%) formed by alcohol-dependent patients exhibiting lifetime mood disorder diagnosis and mild impulsivity; *Class 2* (14%) formed mainly by lifetime cocaine use disorder patients with low probability of comorbid mental disorders and mild impulsivity; *Class 3* (10.7%) formed by cocaine use disorder patients with elevated probability to course with lifetime anxiety, early and personality disorders, and greater impulsivity scores; and *Class 4* (29.9%) formed mainly by patients with alcohol and cocaine use disorders, with elevated probability in early and personality disorders and elevated impulsivity. Furthermore, there were significant differences among classes in terms of Diagnostic and Statistical Manual of Mental Disorders-4th Edition-Text Revision criteria for abuse and dependence: *Class 3* showed more criteria for cocaine use disorders than other classes, while *Class 1* and *Class 4* showed more criteria for alcohol use disorders.

**Conclusion:**

Cocaine- and alcohol-addicted patients who were grouped according to diagnosis of substance use disorders, psychiatric comorbidity, and impulsivity show different clinical and sociodemographic variables. Whereas mood and anxiety disorders are more prevalent in alcohol-addicted patients, personality disorders are associated with cocaine use disorders and diagnosis of comorbid substance use disorders. Notably, increased impulsivity is a distinctive characteristic of patients with severe cocaine use disorder and comorbid personality disorders. Psychiatric disorders and impulsivity should be considered for improving the stratification of addicted patients with shared clinical and sociodemographic characteristics to select more appropriate treatments.

## Introduction

Lifetime substance use disorders are characterized by compulsive drug-seeking despite harmful consequences (1). In Europe, substance use disorders are considered complex socio-sanitary problems because they consist of several biological, behavioral and socio-environmental factors (2). Alcohol is the most commonly used psychoactive substance, and its consumption is associated with an important set of heterogeneous clinical features (3) such as the prevalence of comorbid mental disorders, cognitive impairment, alcoholic liver disease, *delirium*, or alcoholic dementia over time (4). In addition to alcohol use disorders, cocaine powder abuse has increased during the last decade, raising the incidence of important clinical complications, mainly in young adults (5, 6) and becoming the second illegal substance consumed in Europe (2). Patients with cocaine use disorders course with an elevated prevalence of mental problems, mainly those related to anxiety and psychotic disorders (7–9), long-term cardiovascular complications and neurological impairments (10, 11).

In clinical practice, it is not difficult to find patients with more than one substance identified as the reason for treatment demand. Most substance use disorder patients have additional comorbid drug use disorders; despite alcohol being considered the primary substance in patients seeking treatment, alcohol use disorder patients usually combine it with other substances such as marijuana, cocaine, and/or sedatives (12, 13). Cocaine is one of the most illicit drugs co-occurring with alcohol use, with a prevalence of more than half of outpatient cases (14, 15). Cocaine and alcohol combination potentiates cocaethylene formation, enhancing the cardiotoxic and neurotoxic effects of cocaine or alcohol alone, and the additive effects of muscarinic blockade and sympathetic stimulation (16, 17).

Notably, patients seeking treatment for cocaine and alcohol use disorders are frequently affected by lifetime psychiatric comorbidity, mainly mood disorders, anxiety, psychosis, and personality disorders (9, 18–20). In fact, the diagnosis of various substance use disorders in the same subject (e.g., cocaine and alcohol) is associated with elevated addiction severity, high prevalence of comorbid mental disorders, and difficulty in maintaining abstinence. Consequently, more relapses have been reported in these addicted patients with psychiatric complications (comorbid mental and substance use disorders) (15, 21).

Personality constructs have long been associated with addictions, and impulsivity has been considered important in the psychiatric comorbidity of addiction (22, 23). Impulsivity is not a single construct, as it comprises several aspects such as impulsive choice (preference for immediate over delayed rewards), impulsive actions (the possibility of inhibition of a motor response), and impulsive personality traits (the self-regulatory capacity) (24, 25). Dysfunctional impulsivity has been noticed in the compulsive seeking and loss of control over drug intake, as well as withdrawal symptoms (26–28). Therefore, impulsivity is a feature that must be considered for the etiology, course, and development of substance use disorders and their psychiatric complications (29, 30).

The major challenge facing mental health and addiction treatment services is elevated relapse rates, even after long periods of substance abstinence, particularly in outpatient programs (15). Currently, a growing body of clinical studies in addicted populations is focused on identifying potential biomarkers related to substance use disorders (addiction severity, psychiatric comorbidity, etc.) and improving the stratification of patients for the use of appropriate behavioral and pharmacological therapies (19, 20, 31).

In this study, we explored the hypothesis that psychiatric comorbidity and impulsivity are relevant clinical variables, which might help in the stratification of addicted patients to improve the efficacy of treatments. The aim of this study is to identify and characterize heterogeneous subgroups of cocaine- and alcohol-addicted patients with common phenotypic features using a latent class analysis (LCA) (32, 33) with lifetime substance use disorders, psychiatric comorbidity, and impulsivity as primary variables on account of their link to the etiology and severity of substance use disorders.

## Materials and Methods

### Participants and Recruitment

We evaluated a sample of 214 white Caucasian participants recruited from different outpatient setting programs in active treatment for lifetime cocaine and/or alcohol use disorders in the province of Málaga (Spain) and at *Hospital Universitario 12 de Octubre* (Madrid, Spain).

The participation in the study was voluntary and had to meet eligibility based on inclusion and exclusion criteria. The inclusion criteria included ≥18 up to 65 years of age, lifetime cocaine and/or alcohol diagnosis, and at least 2 weeks of abstinence. The exclusion criteria were the diagnosis of other substance use disorders (distinct to cocaine and alcohol use disorders), and the presence of cognitive incapacity to complete the clinical assessments and the self-reported evaluation.

### Demographic Characteristics and Clinical Assessments

All participants were assessed for sociodemographic variables and were administered a comprehensive clinical battery by trained research staff.

Before the clinical assessment, the “Trail Making Test” Part B was easily administered to all participants as a memory and attention-screening test to detect cognitive alteration (34). Participants were evaluated using the Spanish version of the “Psychiatric Research Interview for Substance and Mental Diseases” (PRISM) according to “Diagnostic and Statistical Manual of Mental Disorders-4th Edition-Text Revision” (DSM-IV-TR) criteria (35, 36). The semi-structured interview PRISM is designed expressly to assess comorbid psychiatric disorders in individuals with drug use. Lifetime substance use prevalence was applied to measure the frequency of cocaine and alcohol use disorders, distinguishing abuse from dependence symptoms. In addition, the four criteria for abuse and the seven criteria for dependence were included as a quantitative indicator according to DSM-IV-TR for symptom severity. This one-dimensional score is in agreement with DSM-5 criteria [for more details, see Ref. (9, 19, 20, 37)]. For mental comorbidity associated with substance use disorders, we evaluated mood, anxiety, psychotic, eating, personality (borderline and antisocial personality disorders), and early-onset disorders in childhood [conduct disorder and attention deficit hyperactivity disorder (ADHD)]. All diagnoses were classified taking lifetime prevalence (absence/presence) into account.

### Self-reported Impulsivity

To evaluate the cognitive impulsivity constructs, we used the Spanish version of the UPPS-P Impulsive Behavior Scale [abbreviated from negative Urgency, Premeditation (lack of), Perseverance (lack of), Sensation seeking, and Positive urgency (38, 39)] designed to measure five distinct constructs of impulsivity and a total score. This scale is a 59-item measure of impulsive personality traits with five subscales: negative urgency (α = 0.87); lack of premeditation (α = 0.85); lack of perseverance (α = 0.87), and positive urgency (α = 0.93) (25); and each item of the UPPS-P scale is rated on a 4-point scale ranging from strongly agree to strongly disagree. Both UPPS-P and original scale (UPPS scale) have proven useful in characterizing impulsive behavior related to the more common diagnostic in the DSM-IV, including substance use disorders [alcohol use dependence (40) and cocaine dependence (41, 42)] or pathological gambling (43). Furthermore, UPSS-P scale is considered an effective instrument in clinical and research contexts based on its psychometric properties (39).

### Statistical Analysis

Correlation analyses were performed using the Pearson’s correlation coefficient (*r*) followed by the Bonferroni correction test for multiple comparisons were used to explore the relationship between impulsivity measures in the UPPS-P scale and the DSM-IV-TR criteria for abuse and dependence.

An LCA was performed to identify different clusters based on multiple and heterogeneous variables from our sample. LCA is a probabilistic technique that assigns individuals to classes (44, 45) and was conducted using Latent Gold 4.5 software (Statistical Innovations, Belmont, CA, USA). Dichotomous indicators (absence/presence) were created for lifetime diagnoses and included in LCA along with the UPPS-P total score. Thus, LCA was performed to identify latent classes (LCs) based on the probability of individuals belonging to different clinical profiles defined by these variables. The best-fitting model was selected based on quantitative and qualitative preponderance of evidence. Goodness-of-fit quantitative statistics indicators included the Akaike Information Criterion (AIC) and Bayesian Information Criterion (BIC). Lower AIC and BIC values indicated better balance model parsimony and model fit. The Bootstrapped Likelihood Ratio Test (BLRT) was used to compare the model with *k* classes to the model with *k* − 1 classes, and significant outcomes (*p* ≤ 0.05) indicated that the model with *k* classes was better fitted to the data. In addition, entropy (a classification quality index) was obtained, with values closer to 1 indicating a better fit. The optimal solution was considered according to the lowest BIC/AIC, the highest entropy, and a significant BLRT value.

Differences among LCs in demographic–clinical variables and impulsivity were assessed using chi-square test for categorical variables and one-way analysis of variance (ANOVA) for continuous variables. In addition, an analysis of covariance (ANCOVA) was performed to discard the possible effect of age as a covariate of UPPS-P scores. Both ANOVA and ANCOVA were followed by *post hoc* Newman–Keuls tests. Only significant results (*p* ≤ 0.05) are shown.

## Results

### Sociodemographic Characteristics and Substance Use Disorders

Table 1 shows a sociodemographic description of the sample. The average participant was a 43-year-old male with a secondary educational level (49%), mostly unemployed (51%), and a previous history with outpatient treatment for addiction (56%).

**Table 1:** Sociodemographic variables.

Variable		Total *N* = 214
**Age *[mean (SD)]***	Years	43.4 (10.5)
**Sex *[N (%)]***	Women	48 (22.4)
	Men	166 (77.6)
**Marital status *[N (%)]***	Never married	81 (37.9)
	Married/cohabitating	73 (34.1)
	Divorced/separated	57 (26.6)
	Widowed	3 (1.4)
**Educational level *[N (%)]***	No studies	5 (2.3)
	Primary/elementary	75 (35)
	Secondary	104 (48.6)
	University	30 (1.4)
**Work status *[N (%)]***	Unemployed	109 (50.9)
	Employed	92 (43)
	Retired	13 (6.1)
**Criminal record *[N (%)]***		75 (35)
**Medical history *[N (%)]***	Outpatient	119 (55.6)
	Hospitalized	8 (3.7)
	Both	48 (22.4)

The cohort was recruited from outpatient programs for both alcohol (*N* = 136) and cocaine (*N* = 78) treatments, and the prevalence of substance use disorders is indicated in Table 2. While we found that 32.4% of alcohol-addicted patients were diagnosed with comorbid cocaine use disorders, 53.8% of patients in treatment for cocaine use were diagnosed with comorbid alcohol use disorders. Therefore, addicted patients were diagnosed with cocaine abuse (48.6%), cocaine dependence (44.4%), alcohol abuse (77.1%), and alcohol dependence (75.7%).

**Table 2:** Prevalence of substance use disorders in patients treated for alcohol and cocaine.

Substance use disorders	Total *N* = 214
**Treatment for alcohol use *[N (%)]***	136 (63.6)
**Alcohol use disorders**	136 (100)
Abuse	124 (91.2)
Dependence	131 (96.3)
**Cocaine use disorders**	44 (32.4)
Abuse	43 (31.6)
Dependence	28 (20.6)
**Treatment for cocaine use *[N (%)]***	78 (36.5)
**Cocaine use disorders**	78 (100)
Abuse	61 (78.2)
Dependence	67 (85.9)
**Alcohol use disorders**	42 (53.8)
Abuse	41 (52.6)
Dependence	31 (39.7)

### Comorbid Mental Disorders and Impulsivity

In the total sample, there was an elevated prevalence in comorbid mental disorders, particularly mood disorders (37.9%), early-onset disorders (32.7%), personality disorders (32.2%), and anxiety disorders (18.7%). However, the prevalence of these mental disorders was different according to each substance use disorder. As shown in Table 3, mood disorders were more prevalent in patients with alcohol use disorders (alcohol abuse and dependence) but chi-square test revealed no significant differences. By contrast, personality and early-onset disorders were significantly more prevalent in cocaine use disorders (*p* < 0.01).

**Table 3:** Comorbid mental disorders and impulsivity in addicted patients according to substance use disorders.

Total *N* = 214		Cocaine abuse	Cocaine dependence	Alcohol abuse	Alcohol dependence	*p*-Value
Patients [*N* (%)]		104 (48.6)	95 (44.4)	165 (77.1)	162 (75.7)	
Comorbid mental disorders [*N* (%)]	Mood	35 (33.0)	29 (30.5)	67 (40.6)	69 (42.6)	0.171^a^
	Anxiety	24 (23.1)	21 (22.1)	33 (20.0)	31 (19.1)	0.850^a^
	Psychotic	14 (13.5)	12 (12.6)	19 (11.5)	18 (11.1)	0.405^a^
	Eating	3 (2.9)	4 (4.2)	3 (1.8)	2 (1.2)	0.438^a^
	Personality	49 (47.1)	46 (48.4)	55 (33.3)	49 (30.2)	0.003^a^
	Early-onset	54 (51.9)	46 (48.4)	56 (33.9)	50 (30.9)	0.001^a^
Impulsivity [mean (SD)]	UPPS-P total score (range: 59–236)	150.00 (22.46)	150.69 (21.85)	143.21 (22.60)	142.29 (22.17)	0.003^b^
	Lack of premeditation (range: 11–44)	28.19 (7.43)	28.52 (7.24)	28.64 (8.00)	28.91 (8.23)	0.907^b^
	Sensation seeking (range: 12–48)	32.42 (9.03)	32.55 (8.47)	29.68 (9.78)	29.28 (9.76)	0.006^b^
	Lack of perseverance (range: 10–40)	21.29 (6.30)	20.98 (6.39)	20.10 (6.21)	19.65 (5.73)	0.120^b^
	Negative urgency (range: 12–48)	32.87 (7.22)	32.65 (7.31)	31.86 (7.47)	32.09 (7.54)	0.675^b^
	Positive urgency (range: 14–56)	35.26 (9.82)	36.00 (9.54)	32.93 (9.87)	32.37 (9.65)	0.008^b^

*^a^p-Value from chi-square test*.

*^b^p-Value from analysis of variance*.

In addition to mental disorders, impulsivity was also assessed, and patients with cocaine use disorders showed significantly higher UPPS-P total score (*F*_3,522_ = 4.82, *p* < 0.01), sensation seeking (*F*_3,522_ = 4.25, *p* < 0.01), and positive urgency (*F*_3,522_ = 4.00, *p* < 0.01) than patients with alcohol abuse or dependence.

### Correlation between Substance Use Disorders and Different Impulsivity Subscales

Correlation between DSM-IV-TR criteria for substance use disorders and scores of different subscales of impulsivity are represented in Table 4. This table includes the results of a correlation analysis between criteria for cocaine and alcohol abuse and dependence and the impulsivity subscales. The DSM-IV-TR criteria for cocaine abuse and dependence correlated positively with the higher UPPS-P total score. In addition, criteria for cocaine dependence correlated with the sensation-seeking and positive urgency subscales. By contrast, the DSM-IV-TR criteria for alcohol use disorders showed no significant relationship with impulsivity.

**Table 4:** Correlation between criteria for substance use disorders and impulsivity subscales (UPPS-P).

Variables	Total UPPS-P (score)		Lack of premeditation (score)		Sensation seeking (score)		Lack of perseverance (score)		Negative urgency (score)		Positive urgency (score)	
					
	*r*	*p*-Value	*r*	*p*-Value	*r*	*p*-Value	*r*	*p*-Value	*r*	*p*-Value	*r*	*p*-Value
Alcohol abuse (criteria)	0.047	0.493	−0.066	0.338	0.023	0.734	0.033	0.626	0.077	0.264	0.061	0.377
Cocaine abuse (criteria)	0.266	<0.001	−0.075	0.273	0.219	<0.001	0.175	0.010^a^	0.151	0.027	0.238	<0.001
Alcohol dependence (criteria)	−0.154	0.023^a^	−0.031	0.650	−0.142	0.037^a^	−0.078	0.256	0.026	0.704	−0.164	0.016^a^
Cocaine dependence (criteria)	0.282	<0.001	−0.075	0.273	0.246	<0.001	0.155	0.022	0.112	0.102	0.291	<0.001

*^a^Significant Pearson’s correlation coefficients (*r*) were considered at *p* ≤ 0.002 according to the Bonferroni correction test*.

### LCA and Four-Class Model

A clustering analysis was examined to characterize different phenotypic traits from the heterogeneous cocaine and alcohol cohort of this study. Model fit statistics for one to five-class models are presented in Table 5. The three-class solution corresponded to the most parsimonious model considering the BIC value, although the AIC value indicated that the best model with the greater entropy was the four-class model. The BLRT suggested that the four-class model provided a better fit with respect to the three-class solution and was not improved by the five-class model. The difference between the BIC value and the three- and four-class models was minimal, and the latter had fewer significant residuals. Therefore, the four-class model was chosen as the optimal solution to describe the sample with a probability of 0.96, which indicated that the individuals were correctly classified in each LC.

**Table 5:** Indices for latent class analysis models with 1–5 latent classes.

Number of classes	Log-likelihood	BIC	AIC	Entropy	*p*-Value (BLRT)
1	−2,106.96	4,278.32	4,237.93	–	–
2	−1,984.35	4,102.85	4,018.70	0.89	<0.001
3	−1,940.41	4,084.72	3,956.82	0.89	<0.001
4	−1,917.11	4,107.90	3,936.23	0.91	<0.001
5	−1,905.55	4,154.54	3,939.11	0.83	0.120

*BIC, Bayesian Information Criterion; AIC, Akaike Information Criterion; BLRT, Bootstrapped Likelihood Ratio Test*.

**Figure 1:**
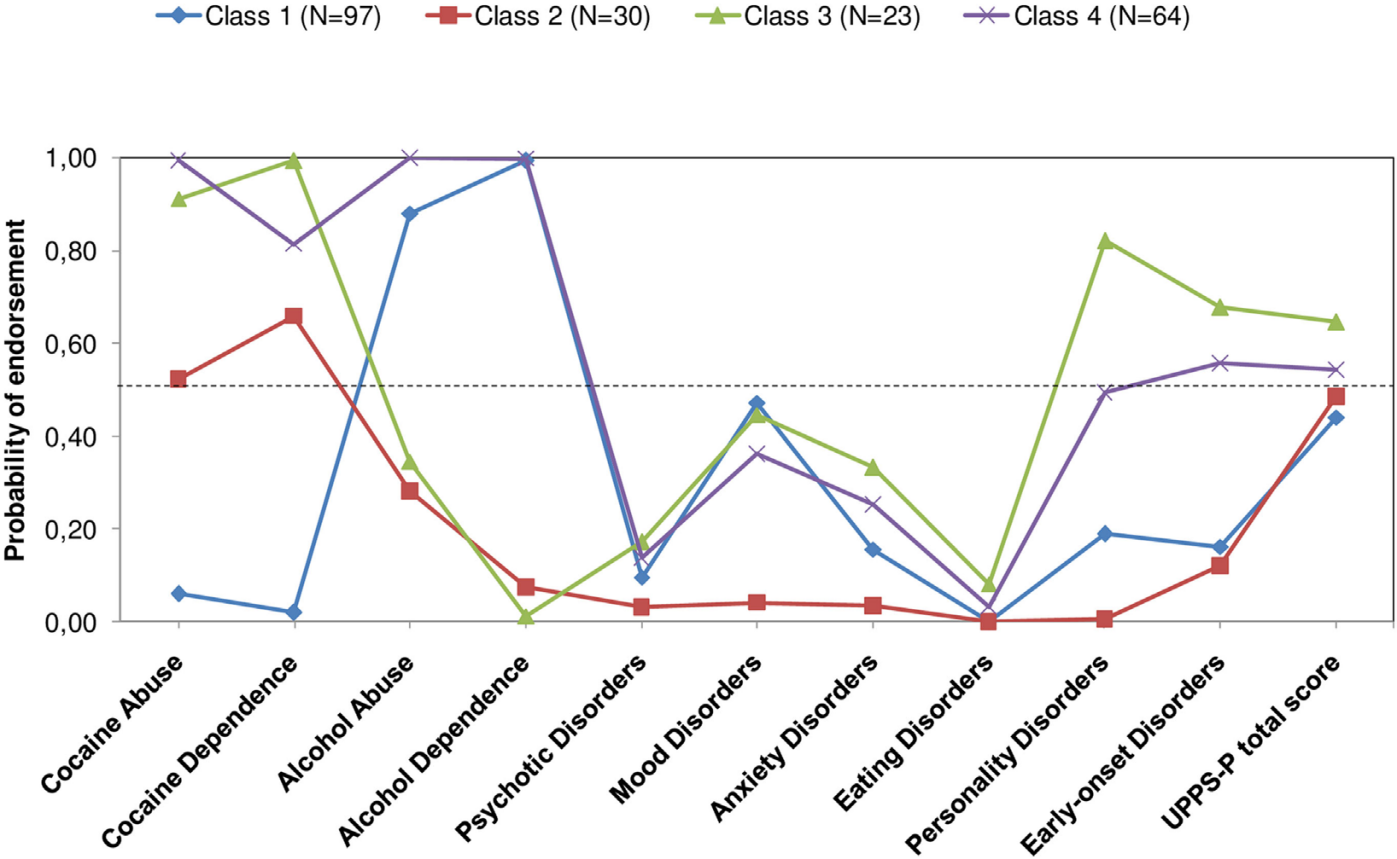
Test performance of LCA-derived analysis. LCA revealed four LCs underlying sample data, with different clinical profiles according to the probability of showing lifetime diagnoses of substance use disorders, comorbid mental disorders, and high impulsivity scores in the UPPS-P scale. Abbreviations: BIC, Bayesian Information Criterion; AIC, Akaike Information Criterion; BLRT, Bootstrapped Likelihood Ratio Test; LC, latent class; LCA, latent class analysis.

The estimated probability for each indicator in the model of four LCs is shown in Figure 1. *Class 1* (45.3% of the sample) was characterized by patients with a high probability of showing lifetime alcohol use disorders, as well as a moderate probability of having a comorbid mood disorders. *Class 2* (14%) included patients with a moderate probability of having cocaine use disorders and a low probability of having a comorbid mental disorder. *Class 3* (10.7%) was composed of patients with a high probability of showing cocaine use disorders, and both early-onset and personality disorders. Finally, *Class 4* (29.9%) included individuals with a high probability of having been diagnosed with both cocaine and alcohol use disorders, with a moderate probability of having comorbid early-onset and personality disorders.

The four LCs showed different sociodemographic variables (Table 6), and the statistical analysis of these variables resulted in significant differences (age, sex, marital status, work status, criminal record, and medical history) except for educational level.

**Table 6:** Sociodemographic variables in addicted patients according to latent classes.

Variables		Total *N* = 214	LC1 (*N* = 97)	LC2 (*N* = 30)	LC3 (*N* = 23)	LC4 (*N* = 64)	*p*-Value
**Age *[mean (SD)]***	Years	43.4 (10.5)	50.3 (7.5)	34.9 (6.9)	32.5 (6.8)	41 (7.84)	<0.001^a^
**Sex *[N (%)]***	Women	48 (22.4)	30 (30.9)	5 (16.5)	5 (21.7)	8 (12.5)	0.042^b^
	Men	166 (77.6)	67 (69.1)	25 (83.3)	18 (78.3)	56 (87.5)	
**Marital status *[N (%)]***	Never married	81 (37.9)	25 (25.8)	15 (50)	14 (60.9)	27 (42.2)	<0.001^b^
	Married/cohabiting	73 (34.1)	44 (45.4)	10 (33.3)	3 (13)	16 (25)	
	Divorced/separated	57 (26.6)	25 (25.8)	–	6 (26.1)	21 (32.8)	
	Widowed	3 (1.4)	3 (3.1)	–	–	–	
**Educational level *[N (%)]***	No studies	5 (2.3)	3 (3.1)	1 (3.3)	–	1 (1.6)	0.434^b^
	Primary/elementary	75 (35)	28 (28.9)	10 (33.3)	13 (56.5)	24 (37.5)	
	Secondary	104 (48.6)	48 (49.5)	16 (56.5)	8 (34.8)	32 (50)	
	University	30 (1.4)	18 (18.6)	3 (10)	2 (8.7)	7 (10.9)	
**Work status *[N (%)]***	Unemployed	109 (50.9)	47 (48.5)	14 (46.7)	16 (69.6)	32 (50)	0.015^b^
	Employed	92 (43)	38 (39.2)	16 (53.3)	7 (30.4)	31 (48.4)	
	Retired	13 (6.1)	12 (12.4)	–	–	1 (1.6)	
**Criminal record *[N (%)]***		75 (35)	26 (26.8)	7 (23.3)	13 (56.5)	29 (45.3)	0.006^b^
**Medical history *[N (%)]***	Outpatient	119 (55.6)	64 (66)	14 (46.7)	8 (34.8)	33 (51.6)	<0.001^b^
	Hospitalized	8 (3.7)	–	–	5 (21.7)	3 (4.7)	
	Both	48 (22.4)	33 (34)	–	1 (4.3)	14 (21.9)	

*^a^*p*-Value from analysis of variance (*F*_3,210_ = 59.3)*.

*^b^p-Value from chi-square test*.

### LCs Differ in Substance Use Disorder Severity

**Figure 2:**
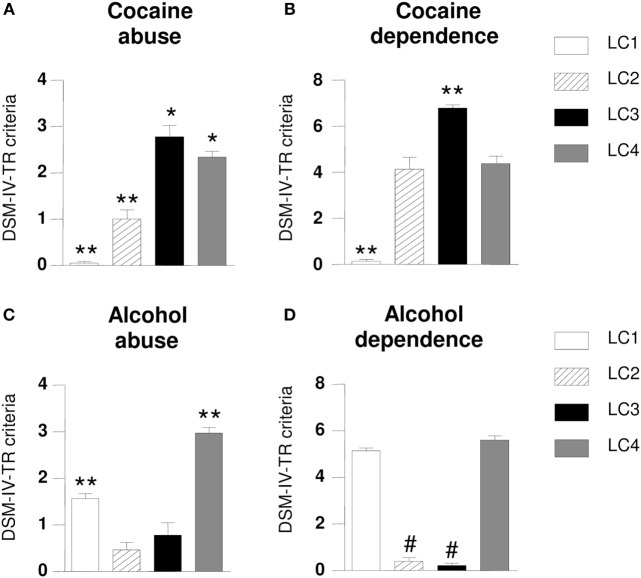
Differences in Diagnostic and Statistical Manual of Mental Disorders-4th Edition-Text Revision (DSM-IV-TR) criteria for substance use disorders according to latent classes (LCs). Abuse diagnosis was based on four DSM-IV-TR criteria, while dependence diagnosis was based on seven criteria. LC3 patients met more cocaine abuse criteria than other classes **(A)**; LC3 patients met more cocaine dependence criteria than other classes **(B)**; LC4 patients met more alcohol abuse criteria than other classes **(C)**; LC4 patients met more alcohol dependence criteria than other classes **(D)**. Average criteria = DSM-IV use disorder criteria. Data are expressed as the mean ± SEM. *Post hoc* Newman–Keuls comparisons: difference vs. the other three groups: *p ≤ 0.05; **p ≤ 0.001; difference vs. LC 1 and LC4: ^#^*p* ≤ 0.001.

The differences among the classes in the broad range of severity of cocaine and alcohol use disorders and impulsivity were measured using one-way ANOVA. Thus, ANOVA revealed differences between classes in the number of DSM-IV-TR criteria for cocaine use disorders (“abuse”: *F*_3,210_ = 90.061, *p* < 0.001; “dependence”: *F*_3,210_ = 125.302, *p* < 0.001) and alcohol use disorders (“abuse”: *F*_3,210_ = 54.063, *p* < 0.001; “dependence”: *F*_3,210_ = 239.082, *p* < 0.001), so that there were differences in terms of severity of these diagnoses. In this regard, individuals in *Class 3* presented more DSM-IV-TR criteria for cocaine abuse and dependence than the other three classes (Figures 2A,B), while *Class 1* and *Class 4* showed more criteria for alcohol abuse and dependence (Figures 2C,D).

### LCs Differ in Impulsivity

**Figure 3:**
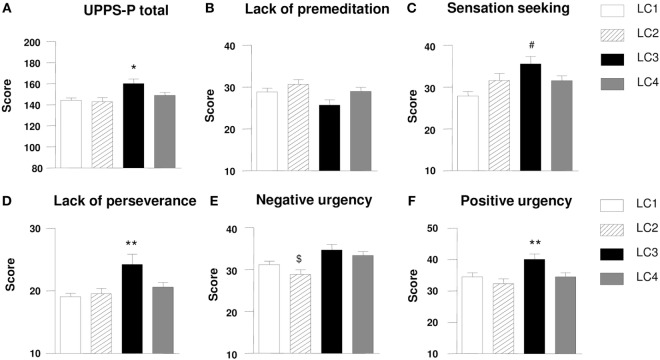
Scores of different impulsivity subscales according to latent classes (LC). LC3 patients showed a greater level of impulsivity than other classes **(A)**; There were no differences between LCs in lack of premeditation **(B)**; LC3 patients showed a greater level of sensation seeking than other classes **(C)**; LC3 patients showed a greater level of lack of perseverance than other classes **(D)**; LC2 patients showed a lower negative urgency in comparison to other classes **(E)**; LC3 patients showed a greater level of positive urgency than other classes **(F)**. Data are expressed as the mean ± SEM. *Post hoc* Newman–Keuls comparisons: difference vs. the other three groups: *p ≤ 0.05; **p ≤ 0.01; difference vs. LC1: ^#^*p* ≤ 0.01; difference vs. LC3 and LC4: ^$^*p* ≤ 0.05.

In addition to psychiatric comorbidity, we evaluated the differences in UPPS-scores according to the different LCs (Figure 3). ANOVA showed that patients belonging to *Class 3* showed greater impulsivity scores in UPPS-P total (*F*_3,210_ = 8.234, *p* < 0.001; Figure 3A); lack of perseverance (*F*_3,210_ = 5.601, *p* < 0.01; Figure 3D) and positive urgency (*F*_3,210_ = 6.838, *p* < 0.001; Figure 3F) relative to the other classes. Lack of premeditation showed no statistical differences between classes (Figure 3B). *Post hoc* comparisons showed that subscale *Class 3* showed a greater score in sensation seeking (*F*_3,210_ = 5.101, *p* < 0.01; Figure 3C) relative to *Class 1*. Therefore, individuals in *Class 2* have significantly lower scores in negative urgency (*F*_3,210_ = 4.178, *p* < 0.01; Figure 3E) compared with those in *Class 3*.

Subsequently, because there was a negative and significant correlation between impulsivity scores in the UPPS-P subscales and age (*r* = − 0.249, *p* < 0.001), a one-way ANCOVA was performed to control the effect of age. The analysis revealed that individuals in *Class 3* obtained significantly higher impulsivity scores in UPPS-total scores (*F*_3,209_ = 4.212, *p* < 0.01), lack of perseverance (*F*_3,209_ = 3.954, *p* < 0.01) and positive urgency (*F*_3,209_ = 3.802, *p* < 0.05), but with no significant differences in sensation seeking and negative urgency subscales (data not shown).

## Discussion

This study reveals that substance use disorders, psychiatric comorbidity and impulsivity allow for the identification of subgroups of addicted patients with common phenotypic features, which has potential clinical relevance. Consequently, the main findings are as follows: (a) Because cocaine- and alcohol-addicted patients seeking treatment show heterogeneous clinical characteristics, the LCA was a statistical tool that identified and established groups based on clinical variables; (b) Substance use disorders, comorbid mental disorders and impulsivity were the most relevant clinical variables to perform LCA in our sample; (c) The four-class model identified four groups of addicted patients with different sociodemographic variables. Our data confirm that impulsivity is an important variable in the context of addiction, particularly with cocaine and alcohol. In fact, a strong correlation was observed between DSM-IV-TR criteria for cocaine use disorders and impulsivity scores, more noticeably with sensation seeking and positive urgency subscales.

The LCA applied to our cohort revealed four different subgroups underlying substance use diagnoses: *Class 1*, individuals who demand treatment for a lifetime of alcohol use disorders with a moderate probability of suffering comorbid mood disorders were characterized by a relative low-profile impulsivity; *Class 2*, those patients with a moderate probability of having cocaine use disorders had a low probability of going through a lifetime comorbid disorder; *Class 3* was composed of patients with cocaine use disorders, which was characterized by elevated impulsivity traits (UPPS-P total, sensation seeking, lack of perseverance and positive urgency) and the highest probabilities of having comorbid personality and early-onset disorders; The last group (*Class 4*) consisted of mixed patients with alcohol and cocaine use disorders with a moderate probability of having comorbid early-onset and personality disorders.

As for sociodemographic characteristics, we found important differences among the LCs. Thus, patients who were diagnosed with cocaine or cocaine and alcohol use disorders (*Classes 2 and 3*) were younger than exclusive alcohol use disorder patients (*Class 1*). Interestingly, *Class 3*, which was composed of individuals with cocaine dependence, showed higher unemployment and criminal rates compared with the other LCs. These results are in accordance with other studies reporting social impairment and other clinical variables (e.g., mood disorders, aggressive behavior, and ADHD) as predictors of substance use disorders (46, 47).

As in our study, comorbid mental disorders have been extensively studied because the prevalence of lifetime mental co-occurrence is salient in the cocaine and alcohol co-user population, especially those related to mood disorders, anxiety disorders, and personality disorders (5, 7, 48). In addition, other authors have shown that cocaine and alcohol co-users highly correlate with psychotic-induced symptoms (18, 49) and with social and emotional impairments (50). Against the existence of a strong relation between substance use and other psychiatric disorders (51), it is known that comorbid mental disorders are linked to impulsive behaviors. Impulsivity has been described as both a risk factor in developing a substance use disorder and as a consequence of substance use (26, 52).

Our findings showed that the *Class 3*, formed mostly by cocaine-addicted individuals, was the most impulsive one (UPPS-P total, sensation seeking, lack of perseverance, and positive urgency) and showed the higher prevalence of personality and early-onset (ADHD) disorders. Although impulsivity is characteristically more frequently present in certain mental disorders such as ADHD or borderline personality (22, 26), we believe that impulsivity is a potential mechanism or a risk factor indicating greater vulnerability to developing cocaine addiction and the appearance of comorbid mental disorders. In fact, we found that the relationship between cocaine severity criteria and impulsivity scores is especially sensitive in the sensation seeking and positive urgency subscales. Sensation seeking has been described as an orientation toward engaging in high energy and thrill behaviors (25). This reward seeking subscale may contribute to the risk, development, and maintenance of substance use disorders, particularly cocaine (53). Regarding positive urgency, this personality trait has been described as the tendency to act immediately in the presence of a positive affect (25) and has been related to sexual risk behaviors and illegal drug use in young adults (54). Despite impulsivity scores showed the highest association with cocaine criteria, we found also an association with alcohol criteria in alcohol-dependent individuals (i.e., UPPS-P total score, sensation seeking, and positive urgency). In accordance with our results, there is an extensive literature linking impulsivity to alcohol use in human studies (30). However, it is important to note that age is a relevant factor linked to impulsivity (e.g., adolescence is critically associated with increased sensation seeking) (55, 56) because age differences are found in the LCs, being *Class 3* the youngest.

Although our findings support the importance of assessing impulsivity in the context of substance use disorders, we are aware of the limitations of a cross-sectional study with a retrospective self-reporting method. First, there are a growing number of social and environmental factors as well as variables associated with addiction (e.g., duration of abstinence) that could influence our data, but these were not included in our analyses (LCA) because of the relatively small sample size, statistical limitations, and differences in sources of recruitment from outpatient programs. Second, this study was conducted in patients diagnosed with cocaine and/or alcohol use disorders, but we have yet to investigate the influence of other substance use disorders (i.e., cannabis) in impulsivity characterization. Third, because only a small number of women seek treatment for addiction, the influence of gender will have to be assessed in larger samples. Finally, all of these limitations will need to be addressed in further research for prevention and therapeutic purposes.

In conclusion, we show that clinical variables associated with the psychiatric health status of a realistic sample of addicted patients could be useful to stratify them to develop appropriate therapies (pharmacological and/or behavioral treatments) according to both clinical and sociodemographic characteristics for each class or group established. The final aim is to improve the treatments and to reduce the high incidence of relapse in outpatient programs of treatment for both cocaine and alcohol use disorders. However, we are aware that the potential implications in clinical practice in the field of addictions will need to be demonstrated in larger samples, taking into account the limitations of the study.

## Ethics Statement

Written informed consent was obtained from each participant after a complete description of the study. All of the participants had the opportunity to discuss any questions or issues. The study and protocols for recruitment were approved by the Ethics Committee of the Hospital Regional Universitario de Málaga (07/19/2009 PND049/2009 and PI0228-2013) in accordance with the “Ethical Principles for Medical Research Involving Human Subjects” adopted in the Helsinki Declaration by the World Medical Association (64th WMA General Assembly, Fortaleza, Brazil, October 2013), Recommendation No. R (97) 5 of the Committee of Ministers to Member States on the Protection of Medical Data (1997), and the Spanish Data Protection Act (Ley Orgánica 15/1999 de Protección de Datos, LOPD). All collected data were given a code number to maintain privacy and confidentially.

## Author Contributions

FF, LS, and EC-O were responsible for the study concept and design. DG-M and FP performed statistical analysis and interpretation of findings. NG-M and AS drafted the manuscript. MP, PA, and NG-M carried out the psychopathological evaluations. GR and JR supervised the clinical recruitment and provided critical revision of the manuscript for important intellectual content. All the authors critically reviewed content and approved final version for publication.

## Conflict of Interest Statement

The authors declare that the research was conducted in the absence of any commercial or financial relationships that could be construed as a potential conflict of interest.
